# A Novel Perspective and Approach to Intestinal Octreotide Absorption: Sinomenine-Mediated Reversible Tight Junction Opening and Its Molecular Mechanism

**DOI:** 10.3390/ijms140612873

**Published:** 2013-06-20

**Authors:** Yuling Li, Zhijun Duan, Yan Tian, Zhen Liu, Qiuming Wang

**Affiliations:** 1 Department of Gastroenterology, First Affiliated Hospital of Dalian Medical University, Dalian 116011, China; E-Mails: lalala5427@163.com (Y.L.); liuzhen0105@163.com (Z.L.); wqming83@163.com (Q.W.); 2 College of Pharmacy, Dalian Medical University, Dalian 116011, China; E-Mail: tiany2004@126.com

**Keywords:** octreotide, sinomenine, intestinal absorption, tight junction

## Abstract

In this work, we assessed the effects of sinomenine (SN) on intestinal octreotide (OCT) absorption both in Caco-2 cell monolayers and in rats. We also investigated the molecular mechanisms of tight junction (TJ) disruption and recovery by SN-mediated changes in the claudin-1 and protein kinase C (PKC) signaling pathway. The data showed that exposure to SN resulted in a significant decrease in the expression of claudin-1, which represented TJ weakening and paracellular permeability enhancement. Then, the recovery of TJ after SN removal required an increase in claudin-1, which demonstrated the transient and reversible opening for TJ. Meanwhile, the SN-mediated translocation of PKC-α from the cytosol to the membrane was found to prove PKC activation. Finally, SN significantly improved the absolute OCT bioavailability in rats and the transport rate in Caco-2 cell monolayers. We conclude that SN has the ability to enhance intestinal OCT absorption and that these mechanisms are related at least in part to the important role of claudin-1 in SN-mediated, reversible TJ opening via PKC activation.

## 1. Introduction

Clinically, the use of hydrophilic peptides has been limited to the parenteral route (intravenous, subcutaneous and deeply muscled injection routes of administration are available [1,2]), because they exhibit low bioavailability upon oral administration [3,4]. Therefore, the burden of injectable drug regimens adversely impacts patients’ quality of life in chronic conditions. For drugs used for long-term treatment or prevention, such as insulin for hyperglycemia in diabetes and octreotide (OCT) for portal hypertension in liver cirrhosis [5], oral administration is the most ideal route. If oral administration could be applied clinically, it would be a safe and convenient method for patients with liver disease to prevent portal hypertension.

As a hydrophilic octapeptide, the paracellular route, which is mainly governed by tight junctions (TJ), is known to be crucial for intestinal OCT absorption [6]. Fortunately, a paper reminded us that sinomenine (SN) could enhance the intestinal absorption of hydrophilic macromolecular drugs, most likely due to a transient opening of the TJs to allow for the paracellular route and/or inhibition of the active drug efflux transport (such as P-glycoprotein, P-gp) for the transcellular route [7]. This finding inspired us to enhance intestinal OCT absorption co-administration with SN and to explore the mechanism of SN-mediated enhancement in intestinal OCT absorption, which remained largely unknown.

In this study, we chose the safe and effective concentration of SN to leading to the changes in transepithelial electrical resistance (TEER) and Fluorescein isothiocyanate dextran 4000 (FD-4) permeability in the Caco-2 cell model. We conducted absorption-enhancing experiments both *in vitro* and *in vivo* to demonstrate the feasibility of OCT oral administration in combination with SN, and we observed the expression of claudin-1 and the activation of the PKC signaling pathway for the paracellular route at the molecular level. Synchronously, we also studied the mechanism of the SN-mediated inhibition of active drug efflux transport along the transcellular route (the mechanisms were submitted elsewhere for publication). Finally, we attempted to investigate the detailed molecular mechanisms of the oral absorption enhancement of OCT using SN. We are looking forward to realizing a general method of oral application for OCT that may then be extended to other peptides.

## 2. Results and Discussion

### 2.1. Everted Gut Sacs Studies in Rats

**Figure 1:**
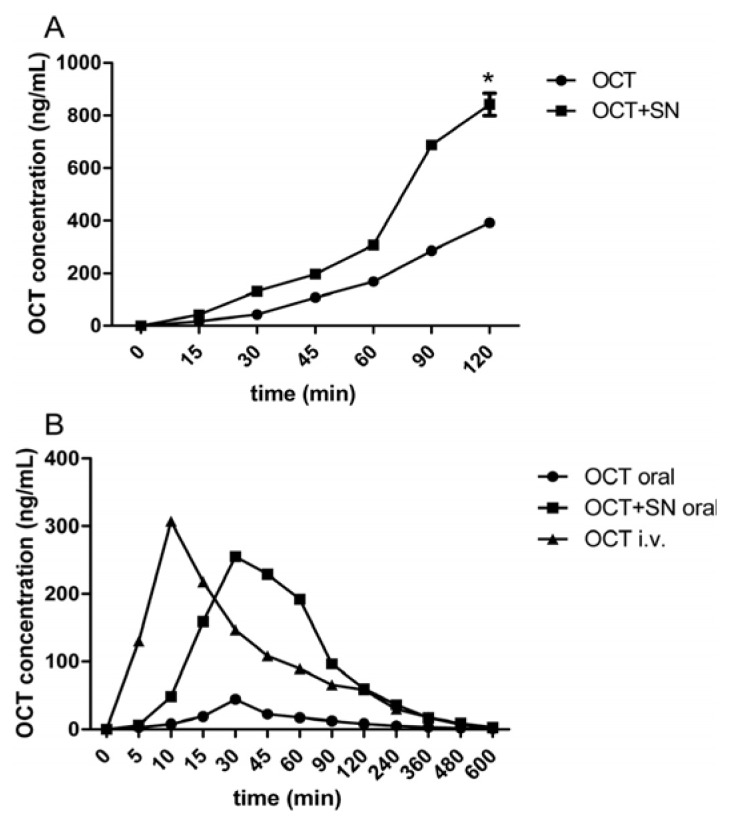
Effect of sinomenine (SN) on the absorption of octreotide (OCT) in rats. (**A**) The everted rat gut sacs experiment of OCT intestinal absorption. The everted rat gut sacs model was performed for 120 min incubation to OCT (10 μM) intestinal absorption with or without SN (0.5% *w*/*v*), which showed a time-dependent increase, * *p* (OCT + SN group *vs*. OCT group at 120 min) < 0.05; (**B**) The i.v. and oral administration of OCT in rats. The rats were orally gavaged with 200 μg OCT with or without SN (30 mg/rat) for oral delivery, then 20 μg OCT for i.v. delivery as contrast. The C_max_ and the AUC were obviously enlarged in OCT + SN group.

To determine the possible effect of SN on the enhancement of OCT intestinal absorption, co-incubated tissue culture tests with 10 μM OCT and 0.5% w/v SN were conducted. As shown in Figure 1A, when treated with 10 μM OCT alone for 120 min, there was good linear correlation between OCT absorption and incubation time (R^2^ = 0.9736). However, when treated with 0.5% *w*/*v* SN and 10 μM OCT together, this correlation became non-linear. After 60 min of incubation, the absorption of OCT significantly increased, and when OCT was co-incubated with SN for 120 min, the absorption of OCT was increased approximately 2.15-fold (* *p* < 0.05, Figure 1). These results suggested that SN enhanced OCT absorption in the intestine.

### 2.2. Intravenous (i.v.) and Oral Pharmacokinetic Studies in Rats

Uptake profiles following i.v. administration of OCT (20 μg/rat) showed a single peak at 10 min (T_max_) with a C_max_ of 307 ng/mL, while the uptake profiles following oral administration of OCT (200 μg/rat) showed a single peak later at 30 min (T_max_), with a C_max_ of 44.3 ng/mL. The oral administration of OCT (200 μg/rat) with SN (30 mg/rat) produced an uptake profile at 30 min (T_max_), with a C_max_ of 255 ng/mL (Table 1, Figure 1B). The absolute bioavailability (F%) of OCT was determined by measuring the area under the uptake curve (AUC) for each delivery method. The AUC of OCT after i.v. administration was determined to be 25135.83 ng/mL/min, and the AUC of OCT following oral administration (OCT alone and the OCT plus SN group) was 4023.33 ng/mL/min and 21268.33 ng/mL/min, with absolute bioavailabilities of 1.63% and 8.67%, respectively. Compared with OCT given alone, the pharmacokinetic parameters C_max_, AUC and F% of OCT co-administered with SN were dramatically improved (* *p*, ^#^*p*, ^$^*p* < 0.05, Table 1). These results indicated that SN significantly enhanced the pharmacokinetic behavior of OCT when they were co-administered.

### 2.3. The Cytotoxic Effect of SN on Caco-2 Cell Monolayers

**Figure 2:**
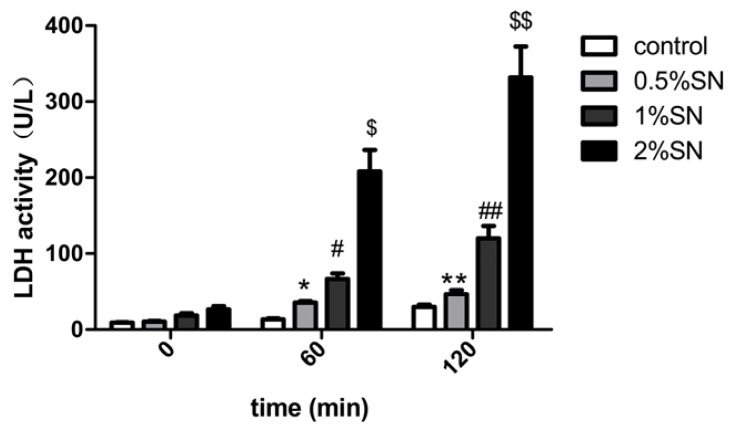
The cytotoxic effect of SN (0.5%, 1%, 2% *w*/*v*) in different concentration on Caco-2 cells. Different concentration of SN (0.5%, 1%, 2% *w*/*v*) were treated on Caco-2 cell monolayers for 120 min incubation. * *p*(0.5% SN treatment group *vs*. control group at 60 min) > 0.05, ^#^*p*(1% SN treatment group *vs*. control group at 60 min) > 0.05, ^$^*p*(2% SN treatment group *vs*. control group at 60 min) < 0.05, ** *p*(0.5% SN treatment group *vs*. control group at 120 min) > 0.05, ^##^*p*(1% SN treatment group *vs*. control group at 120 min) < 0.05, ^$$^*p*(2% SN treatment group *vs*. control group at 120 min) < 0.05.

The cytotoxic effect of different concentrations of SN (0.5%, 1%, 2% *w*/*v*) on Caco-2 cell monolayers was examined using an LDH assay. LDH release was observed in the high (2% *w*/*v*), middle (1% *w*/*v*), low (0.5% *w*/*v*) concentrations of SN and control group (without SN) for 120 min, respectively. The various concentrations of SN exhibited varying degrees of LDH release. Caco-2 cell monolayers exposed to the three concentrations of SN for 120 min had a strong, dose-dependent increase in extracellular LDH activity (Figure 2). After 60 min, only 2% of the SN group showed markedly increased LDH activity (^$^*p* < 0.05, Figure 2), whereas after 120 min, only 0.5% of the SN group did not differ from the control (** *p* > 0.05, Figure 2 ). This result indicated that a 0.5% *w*/*v* concentration of SN was safe for Caco-2 cell monolayers for 120 min of incubation.

### 2.4. Effect of SN on the TEER and FD-4 Permeability of Caco-2 Cell Monolayers

**Figure 3:**
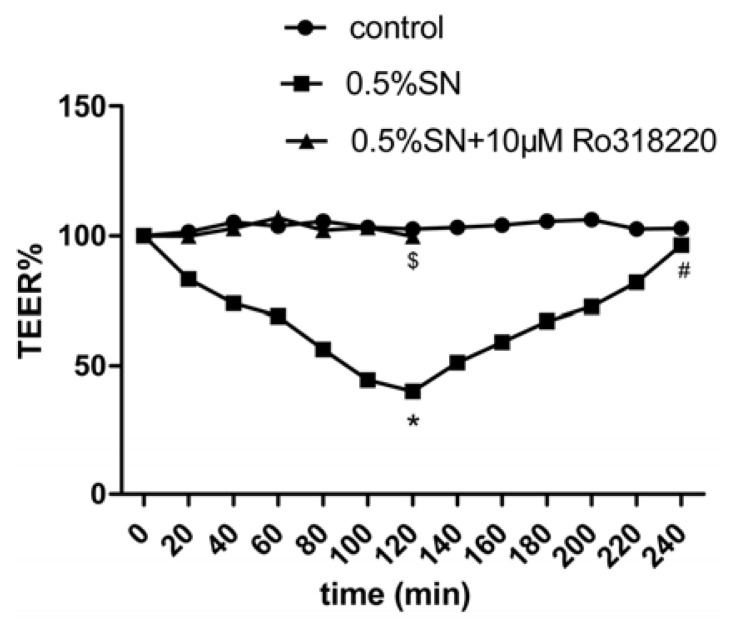
Effects of SN on the TEER (% relative to initial value) of polarized Caco-2 cell monolayers. The lowest point of the TEER% curve appeared at 120 min in 0.5% SN treatment group, which showed the reduce of TEER, an indication of TJs opening between Caco-2 cells. * *p*(0.5% SN treatment group *vs*. control group at 120 min) < 0.05, ^#^*p*(0.5% SN treatment group *vs*. control group at 240 min) > 0.05, ^$^*p*(0.5% SN + 10 μM Ro318220 treatment group *vs*. control group at 120 min) > 0.05.

The integrity of the Caco-2 cell monolayers was evaluated by measuring the TEER, which was used for the transport study and was 600 to 800 Ω·cm^2^. The integrity of the Caco-2 cell monolayers was detected before SN treatment. The TEER values were 746.11 ± 46.57 Ω·cm^2^ (Table 2), indicating good monolayer integrity when subjected to experimentation. Then, the effect of 0.5% SN on the TEER of Caco-2 cell monolayers at pH 7.4 was observed. The percentage TEER relative to the initial TEER values of the Caco-2 cell monolayers after treatment with SN is shown in Figure 3. The 0.5% SN treatment resulted in a strong reduction of TEER (* *p* < 0.05, Figure 3) at 120 min; after SN removal, a gradual recovery in TEER was observed at 240 min, and there was no difference compared with the control (^#^*p* > 0.05, Figure 3). Interestingly, there was no reduction in the TEER values when Caco-2 cell monolayers were treated with 0.5% SN and 10 μM Ro318220 (PKC inhibitor) together at 120 min (^$^*p* > 0.05, Figure 3).

**Figure 4:**
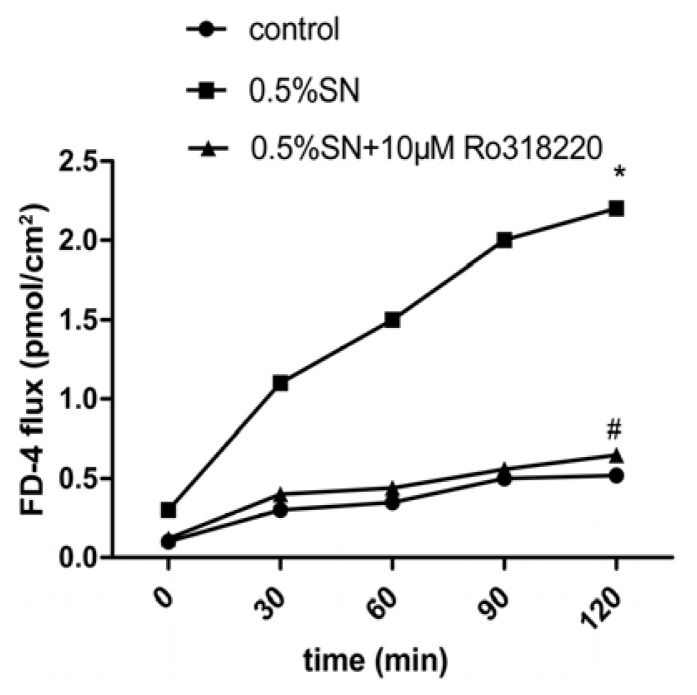
Effects of SN on FD-4 flux in Caco-2 cell monolayers (A-B). Control group means 0.1% FD-4 alone, SN group means 0.5% SN + 0.1% FD-4, SN + Ro318220 group means 0.5% SN + 10 μM Ro318220 + 0.1% FD-4. Permeation clearance of FD-4 was calculated at 120min for each groups. * *p*(SN group *vs*. control group at 120 min) < 0.05, ^#^*p*(SN + Ro318220 group *vs*. control group at 120 min) > 0.05.

The assessment of the potential OCT penetration through TJ was evaluated by measuring FD-4 permeability. A time-dependent increase in FD-4 flux resulting from the SN treatment was observed, with the maximum flux occurring at 120 min (* *p* < 0.05, Figure 4). However, there was not an increase in FD-4 flux, when Caco-2 cell monolayers were treated with 0.5% SN and 10 μM Ro318220 together at 120 min (^#^*p* > 0.05, Figure 4).

These results suggested that the decreased TEER value was associated with an increase in the FD-4 flux and that the TJ were functioning and able to open under SN action in Caco-2 cells; moreover, the opening was reversible after SN removal. Meanwhile, the activation of PKC was related to the observed SN-mediated TJ. The addition of SN induced a decreased systematic TEER value that was associated with an increase in the FD-4 flux rate, which indicates the potential for the penetration of macromolecular drugs TJs, such as OCT.

### 2.5. Effect of Sinomenine on the Transport of OCT across Caco-2 Cell Monolayers

The effect of SN on the transport of OCT across the Caco-2 cell monolayers are summarized in Table 3. The calculated Papp and R values of OCT in the 0.5% SN treatment group is higher than in the OCT alone group (* *p* < 0.05, Table 3 ), which indicated that 0.5% SN significantly increased the transport of OCT in the apical-to-basolateral direction across the Caco-2 cell monolayers at pH 7.4. The results obtained from the OCT transport experiment showed the ability of SN to significantly enhance the transport of this hydrophilic macromolecular drug across intestinal epithelial cell monolayers.

### 2.6. Gene and Protein Levels for the Expression of Claudin-1

**Figure 5:**
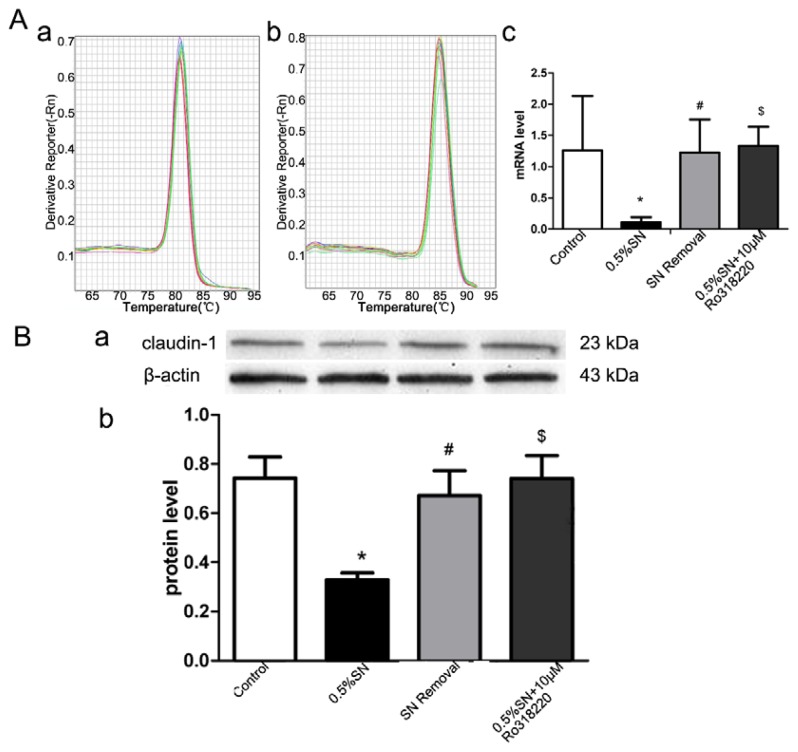

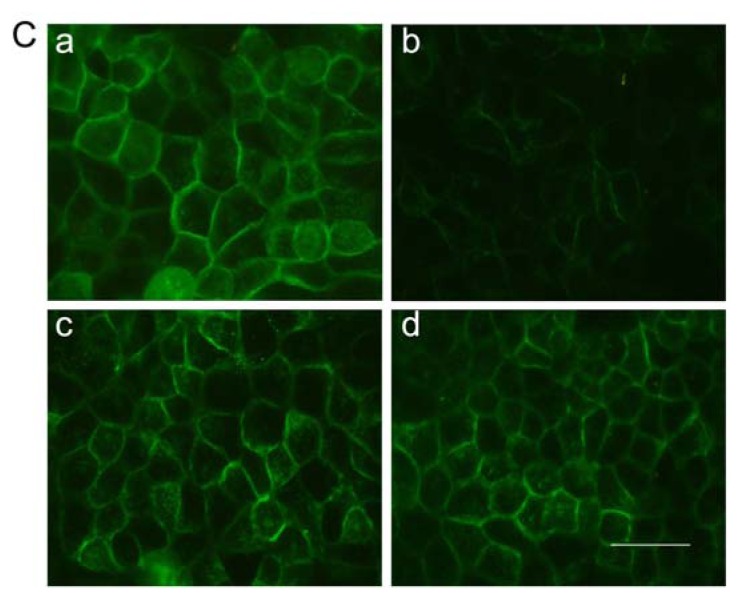
The mRNA and protein levels of claudin-1 expressed in different group. (**A**) The mRNA levels of claudin-1 expressed in different group. (**A-a**), (**A-b**) The melt curve of claudin-1 and β-actin in Real-time PCR, respectively; (**A-c**) Quantitative analysis from Real-time PCR data showed the fold values of the corresponding claudin-1 mRNA to that of β-actin mRNA in control, 0.5% SN treatment, SN removal and 0.5% SN + 10 μM Ro318220 treatment group; (**B**) The protein levels of claudin-1 expressed in different group. (**B-a**) Western bolt data showed the claudin-1 protein expression levels of Caco-2 cell monolayers from control, 0.5% SN treatment, SN removal and 0.5% SN + 10 μM Ro318220 treatment group, respectively; (**B-b**) Quantitative data showed the ratio of band density of the corresponding claudin-1 protein to that of β-actin protein in every group; (**C**) Immunofluorescent staining for localization of claudin-1 protein. Immunofluorescence microscopy for the localization of claudin-1 protein in Caco-2 cell monolayers using Alexa-Fluor^®^488-conjugated Affinipure Goat Anti-Rabbit IgG secondary antibody (green). Fuorescence images collected by inverted microscope. Scale bars: 5 μm. (**C-a**), (**C-b**), (**C-c**), (**C-d**) Immunofluorescent staining of claudin-1 protein in Caco-2 cell monolayers from control (**C-a**), 0.5% SN treatment (**C-b**), SN removal (**C-c**) and 0.5% SN + 10 μM Ro318220 treatment group (**C-d**), respectively. * *p*(SN treatment group *vs*. control group) < 0.05, ^#^*p*(SN removal group *vs*. control group) > 0.05, ^$^*p*(SN + Ro318220 treatment group *vs*. control group) > 0.05.

The quantitative analysis of the gene and protein levels of claudin-1 by SN-induced TJ disruption and their recovery after SN removal were evaluated using real-time PCR and western blotting, respectively. Caco-2 cell monolayers were treated with control, 0.5% SN, SN removal and 0.5% SN together with 10 μM Ro318220 for 2 h, respectively. As shown in Figure 5, after exposure to 0.5% SN for 2 h, a distinct loss of claudin-1 gene (Figure 5A) and protein (Figure 5B) expression were observed in Caco-2 cell monolayers when compared with control (* *p* < 0.05, Figure 5A, B), but after SN removal for 2 h, the expression of claudin-1 returned to its normal level (^#^*p* > 0.05, Figure 5A,B). Strangely, there was no significant difference in the expression of claudin-1, when Caco-2 cell monolayers were treated with 0.5% SN and 10 μM Ro318220 together for 2 h (^$^*p* > 0.05, Figure 5A,B). These results demonstrated that SN treatment could reduce the expression of claudin-1, thus leading to the disruption of TJ in Caco-2 cell monolayers; claudin-1 then returned to its normal level when the SN was washed off. In addition, the SN-mediated changes in claudin-1 might be regulated via the activation of the PKC signaling pathway.

### 2.7. Immunofluorescent Staining for Claudin-1

Caco-2 cell monolayers were treated as described above, and the localization of claudin-1 protein was determined using immunofluorescent staining. Control cells displayed the expected TJ protein localization pattern; claudin-1 was concentrated at the sites of cell-cell contact and formed a belt-like structure, showing continuous and a strong intensity of staining (Figure 5C-a). Treatment with 0.5% SN for 2 h induced the apparent loss of claudin-1 from some distinct membrane regions, indicating the loss of functional TJ from such areas (Figure 5C-b). The removal of SN from the culture medium resulted in a significant recovery of claudin-1 at TJ at 2 h (Figure 5C-c). However, the incubation of the cells with 0.5% SN and 10 μM Ro318220 together resulted in a complete retention of the intensity and staining pattern, as observed in the control group (Figure 5C-d). These findings supported the results of the aforementioned real-time PCR and western blotting.

### 2.8. Mechanism of SN-Mediated Reversible TJ Opening—Shift of PKC-α

**Figure 6:**
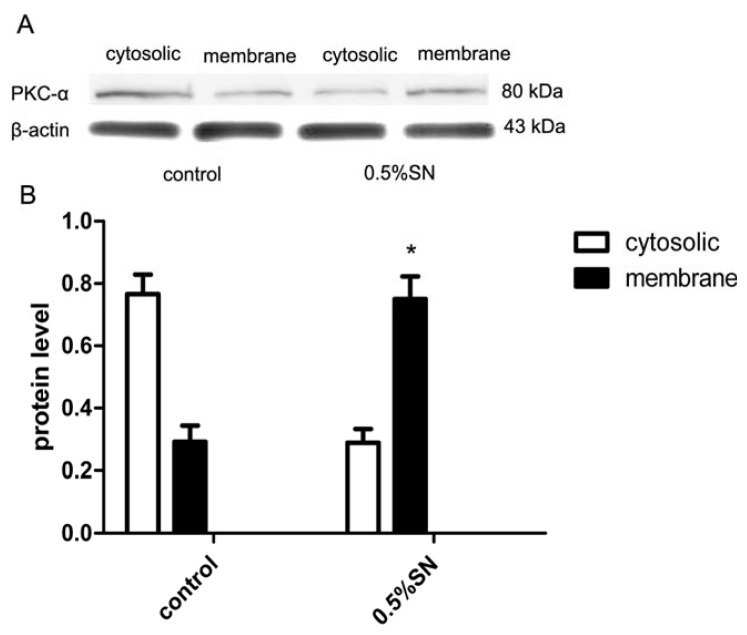
The protein level of PKC-α expressed in different group. (**A**) Western bolt data showed the PKC-α protein expression level in cytosolic and membrane of Caco-2 cell monolayers from control and 0.5% SN treatment group groups, respectively; (**B**) Quantitative data showed the ratio of band density of the corresponding PKC-α protein to that of β-actin in every group. The expression level of PKC-α protein in membrane was increased in 0.5% SN treatment group than in control group (* *p* < 0.05).

We attempted to identify the effect of PKC protein on the disruption of TJ by SN. After 0.5% SN treatment for 2 h, the cytosolic and membrane proteins were extracted from the Caco-2 cells. Control experiments were performed under the same conditions but without SN. The relative content of PKC-α in the cytosolic and membrane fractions of Caco-2 cells were assessed by western blotting. In the control cells, less PKC-α was present in the membrane fraction, however, following treatment with 0.5% SN, most PKC-α was present in the membrane fraction (Figure 6, * *p* < 0.05). This result implied the increased activation of PKC-α following treatment with 0.5% SN. The shift of PKC-α protein from the cytosolic to the membrane fractions is due to the activation of the PKC signaling pathways. The mechanism behind the decrease in claudin-1 protein, which was mediated by SN, was related to the activation of PKC.

### 2.9. Discussion

The population of patients with liver cirrhosis is large, especially in China [8]. OCT is a somatostatin analogue that has few side effects, and its injection is clinically used for lowering the portal hypertension associated with liver cirrhosis [5]. The oral delivery of OCT would have the important clinical significance of avoiding multiple daily injections for these patients. Recently, OCT has attracted a great deal of attention and study to improve its oral delivery. Some of the attempts have reported many encouraging results [3,4,9–11], but to the best of our knowledge, no versions have become commercially available. Previous research has shown that SN could improve the intestinal absorption rate and oral bioavailability of some hydrophilic macromolecular drugs [12–14]. We chose SN as the absorption enhancer of OCT because of its double effects on absorption enhancement and pylic decompression [15].

Our *in vitro* studies, which first examined the effects of SN on the intestinal transport and absorptive kinetics of OCT using the everted rat gut sac system (Figure 1A), indicated that the absorption amount of OCT in the sac contents was markedly elevated by approximately 2.15-fold when treated with 0.5% SN, in comparison with the amount sacs containing OCT alone. The previous literature has revealed that the absorption of hydrophilic macromolecular paeoniflorin was increased approximately 2.5-fold when co-incubated with SN in the everted rat gut sac model, which, in addition to our *in vivo* studies demonstrating the same phenomenon, supported our results [12]. The pharmacokinetic parameters of OCT are summarized in Table 1, and Figure 1B shows that with the co-administration of SN and OCT in rats, both the peak plasma concentration of OCT and the AUC were increased. It is especially noteworthy that the oral co-administration of OCT and SN resulted in a 5.3-fold increase in bioavailability compared with the oral administration of OCT alone. The enhanced pharmacokinetics of the hydrophilic macromolecule paeoniflorin, which were induced by the co-administration of SN, have been similarly documented in previous research [14].

The low absorption via the paracellular route results in an inadequate availability of OCT [6]. A promising approach to enhance the absorption of hydrophilic macromolecular drugs is to co-administer absorption-enhancing agents that reversibly open the paracellular TJ [16]. Our studies in rats indicated that OCT in combination with SN would be orally available. Nevertheless, the mechanism of SN-mediated enhancement in intestinal OCT absorption remains unclear thus far. Therefore, the Caco-2 cell model (a human epithelial cell line originally isolated from a colonic adenocarcinoma, which has been widely accepted as an *in vitro* model of the intestine [17]) was employed to explore this mechanism in our study.

When absorption enhancers are applied in clinical applications, their potential local toxicity should be considered. The membrane damage caused by absorption enhancers is usually estimated using the levels of LDH (a cytosolic enzyme that is released into the culture medium when cells are injured) released from the intestinal epithelium [18]. Based on our LDH test, both the high (2% *w*/*v*) and middle (1% *w*/*v*) concentrations of SN had cytotoxic effects on the Caco-2 cell monolayers, and as a result, we therefore chose the low concentration (0.5% *w*/*v*) of SN for our research (Figure 2).

The TEER value and FD-4 permeability are common measures of paracellular permeability in Caco-2 cell monolayers [19]. SN can create a decrease in TEER and an increase in FD-4 permeability [7]. The percentage of TEER relative to the initial value after 120 min of incubation decreased to 43% with SN in our study (Figure 3). The opening of TJs between adjacent epithelium, as indicated by a reduction in TEER, is often used to predict an ability to enhance drug transport across the epithelial cell monolayers via the paracellular route [7]. Next, a rapid and complete recovery of the TEER values 2 h after the removal of SN was observed (Figure 3). The completely reversible effect of SN on the TEER of Caco-2 cell monolayers demonstrated that no damage occurred to TJ or the epithelial cells [7]. In the meantime, when TEER dropped to nadir after a 120 min of incubation with SN, the permeability of FD-4 reached its highest level (Figure 4), which provided the evidence for the absorption-enhancing effect on OCT. Based on these studies, a transient and reversible opening of TJs seems less damaging than a disruption of the cell membrane structure [20]. Our test showed that SN resulted in a transient and reversible opening (after just 2 h) of TJ, with a potential for the increased paracellular movement of OCT across the cell monolayers. Any other absorption-enhancing agents (such as Chitosan, which requires 24 h to reversibly open TJ [21]) cannot achieve similar results. The transient and reversible opening of TJs in intestinal epithelial cells not only significantly increases the transport of OCT via the paracellular route but also it should reduce the occurrence of spontaneous bacterial peritonitis that may be caused by the sustained TJ opening when SN and OCT further used for animal and human therapy.

The transport of OCT across Caco-2 cells was also evaluated using SN (Table 3). The *P_app_* values of OCT were much higher in SN treated cells than in control cells, and this variation may indicate that SN was able to enhance the transport of OCT. In addition, the calculated *P_app_* and *R* values obtained from the insulin transport study in a previous report also showed the ability of SN to enhance the transport of a hydrophilic macromolecular compound across intestinal epithelial cell monolayers [7].

TJ are dynamic protein structures that form a regulated barrier for movement of molecules through the intercellular spaces across the intestinal epithelium. The discovery of the transmembrane proteins of TJs resulted in great advances in disease therapies, drug discovery and drug targeting [22]. Claudin-1 is the major component of contributing to the integrity of TJ [23,24]. The alteration of claudin-1 mediated by SN was first evaluated by real-time PCR at the gene level and was further examined at the protein level. The level of claudin-1 gene in Caco-2 cells was minimal after exposure to SN for 2 h and then returned to the normal level 2 h after SN washed off (Figure 5A). The expression level of the claudin-1 protein obtained in western blot analysis (Figure 5B) correlated well with the trend in its gene level. Immunofluorescent staining for the localization of claudin-1 also confirmed PCR and western blot (Figure 5C). The aforementioned data suggested that claudin-1 played a significant role in the SN-mediated changes of TJs. At least in part, SN could reversibly regulate claudin-1 and open the TJs of intestinal epithelial cells; however, Pinton [25] deduced that the presence of claudin alone in the TJs may not have been sufficient to achieve a paracellular seal in the intestinal epithelial cells. This finding means that other proteins in TJ also play an important role in maintaining intestinal permeability and that it is essential to determine the identity of the other relevant proteins in future studies.

The dynamic changes, including the disintegration and reassembly of TJ, are regulated by signaling pathways in epithelial barriers [24,26]. In addition, the protein kinase C (PKC)-dependent signaling pathway appears to be of key importance [26,27]. We found that the PKC inhibitor Ro318220 prevented the SN-mediated decrease in TEER (Figure 3) and increase in FD-4 permeability (Figure 4), which preliminarily proved that the SN-mediated impact on TJs was most likely due to PKC activation. This finding was further investigated using western blot analysis. We then observed Ro318220 prevent the SN-mediated decrease in the expression of claudin-1 at both the gene (Figure 5A) and protein levels (Figure 5B,C), which was in addition to the changes in TEER. Ro318220, which is known to inhibit all classical and novel PKC isozymes by competing for the ATP binding site present on the PKC protein, was sufficient to prevent the PKC-mediated events [27,28]. All of these results hinted at the fact that the mechanism of SN-mediated TJs is likely related to a PKC-dependent signaling pathway.

The shift of PKC from the cytoplasm to the plasma membrane indicates that the activation of PKC [24,29], and conventional PKC isoforms (α,β,II) participate in TJ opening or disassembly [24]. To elicit the activation of the PKC signaling pathway, we attempted to investigate the relative content of PKC-α in the cytosolic and membrane fractions of Caco-2 cell monolayers by western blot analysis. In control cells, most of the PKC-α was present in the cytoplasm. However, following SN treatment for 2 h, the PKC-α was more obviously present in the membrane (Figure 6). TJ opening is accompanied by the translocation of cPKC-α from the cytoplasm to the plasma membrane [27]. This result supported the hypothesis that SN mediates changes in TJ integrity at least in part by up-regulating PKC-α activity. However, how this SN-mediated activation augments PKC is currently not understood. It is possible that SN activates specific cell surface receptors, thus leading to the activation of PKC-dependent signaling pathways. Determining whether the other signaling pathways participate in the regulation of TJs is essential for further studies.

The results above indicated that SN is capable of enhancing the intestinal OCT absorption, which is at least in part related to the mechanism of TJ protein claudin-1 and the PKC signaling pathway for the paracellular route. SN was also reported to inhibit active efflux transport for a P-gp substrate for the transcellular route [7,12–14,18]. Meanwhile, our prophase experiment showed that OCT is a P-gp substrate. Therefore, SN could also influence the intestinal absorption and transport of OCT, which were modulated by the P-gp efflux transporter.

In consideration of the decompression effect of SN [15], SN has been identified as a potential safe and effective absorption enhancer of OCT for portal hypertension due to liver cirrhosis. This study provided the theoretical basis for improving the oral bioavailability of OCT. The oral delivery of OCT in combination with SN has significant clinical potential as a novel, non-invasive approach to the treatment of liver cirrhosis.

However, it is important to note that TJ disruption likely occurs through multiple mechanisms and that the effect of certain pathways greatly differs among tissues, cell types, and models. We completed this study only in rats using the Caco-2 cell model, so it is difficult to draw general conclusions related to the absorption enhancing mechanism of SN. Though this study has contributed to advances in enhanced drug delivery through the paracellular pathway, many challenges still need to be overcome before more clinically successful formulations can be produced. We are looking forward to achieving a general method of oral administration for OCT and to extending this knowledge to the administration of other peptides.

## 3. Experimental Section

### 3.1. Materials

Octreotide (OCT, *M*_w_ 1019.26 kDa) was obtained from Chengdu Xinlibang Bio-pharmaceutical Co., Ltd., (Chengdu, China). Sinomenine hydrochloride (SN) was obtained from Nanjing Zelang Pharmaceutical Co., Ltd., (Nanjing, China). Ro318220 (PKC inhibitor) was obtained from Abcam (Cambridge, MA, USA). Fluorescein isothiocyanate dextran 4000 (FD-4, *M*_w_ 3850 kDa) was purchased from Sigma Chemical Co. (St. Louis, MO, USA). All other chemicals were of analytical grade and were used as received.

### 3.2. Animals Ethics

The experimental protocols were approved by the Animal Care and Use Committee of Dalian Medical University (Dalian, China) in accordance with the Code of Ethics of the World Medical Association.

### 3.3. Methods

#### 3.3.1. Everted Gut Sacs in Rats

This procedure was performed as described previously [30]. Male Sprague-Dawley (SD) rats weighing 250–300 g were divided into either the OCT group or the OCT plus SN group. The rats were fasted for 24 h prior to the experiment but were allowed free access to water. They were then sacrificed by cervical dislocation, and the jejunum was removed and washed through three times with saline (0.9% NaCl solution) at room temperature. The jejunum was immediately placed into 37 °C oxygenated (O_2_/CO_2_, 95%:5%) Krebs-Ringer’s buffer (KRB). The jejunum was everted on a glass rod (0.5 cm in diameter), and one end was clamped; then, the small sacs (5 cm in length) were tied using silk braided sutures and fixed onto a sample connection before filling with KRB. Then, each sac filled with KRB was placed in a 10-mL Eppendorf tube containing 7.0 mL of oxygenated KRB at 37 °C, which contained 10 μM OCT with or without 0.5% *w*/*v* SN. Samples of 50 μL were withdrawn at time intervals of 5, 10, 15, 30, 45, 60, 90 and 120 min from each sac. The samples withdrawn were replaced with an equal volume of KRB.

The samples were stored at −20 °C until Liquid Chromatogram-tandem mass spectrometry (LC-MS/MS, HP1200, Agilent, Minneapolis, MN, USA) analysis.

#### 3.3.2. Pharmacokinetics of OCT after i.v. and Oral Administration

After 24 h of fasting, the rats were orally gavaged with 200 μg OCT dissolved in 2 mL saline (0.9% NaCl solution) with or without SN (30 mg/rat) for oral delivery. For i.v. delivery, 20 μg OCT was dissolved in 1 mL saline (0.9% NaCl solution). At time zero (0 min), OCT was delivered to each rat, and the SD rats were then anesthetized. A thin silicone and heparinized cannula was inserted into the jugular vein for blood sample collection. At 5, 10, 15, 30, 60, 90, 120, 240, 360, 480 and 600 min after OCT delivery, 0.3-mL blood samples for the designated time period of each rat were exsanguinated from the jugular vein cannula; then, 1 mL heparinized physiological saline was given back to fill the dead volume of the cannula and avoid blood clotting.

Blood samples were collected in heparinized Eppendorf tubes, placed directly on ice and then centrifuged for 10 min at 5000 rpm at 4 °C. The plasma obtained was stored at −20 °C until LC-MS/MS analysis.

The pharmacokinetic parameters were calculated according to the literature [31]. The AUC was calculated using the linear trapezoidal rule. This value represents the total extent of OCT absorption into the systemic circulation or the total uptake following its administration. The absolute bioavailability values after the oral administration of OCT were calculated using the following formula: F = (AUC_oral_ × D_IV_)/(AUC_IV_ × D_oral_) × 100%, where F is the absolute bioavailability and D is the administered dose. The absorption enhancement ratios were calculated using the following formula: ER = F(OCT + SN)/F(OCT alone).

#### 3.3.3. Cell Culture

Our Caco-2 cells, obtained from the Chinese Academy of Medical Sciences, were cultured in high glucose Dulbecco’s modified eagle’s medium (DMEM, Gibco, Bethesda, MD, USA), supplemented with 10% fetal bovine serum (Gibco), 0.5 U/mL penicillin and 0.1 mg/mL streptomycin. Cells were cultured in a humidified atmosphere of 5% CO_2_ at 37 °C. The growth medium was changed every 2–3 days and was subcultured at 80% confluence by trypsinization. The cells were used for experimental purposes between passages 45 and 55.

#### 3.3.4. Lactate Dehydrogenase Assay

To examine the toxic effects of SN to the Caco-2 cells, a lactate dehydrogenase (LDH) release assay was performed according to Madara and Stafford [32]. The Caco-2 monolayer, which had been cultured in 24-well plates for 21 days, was washed with 500 μL of the PBS and was then incubated with 500 μL of the PBS containing the samples (0.5%, 1%, 2% *w*/*v* SN) to be tested at 37 °C for 120 min. Control experiments were performed under the same conditions but without SN. The amounts of LDH in the supernatant were determined using the enzymatic method and following the manufacturer’s instructions of LDH-Cytotoxic test kit from Nanjing KeyGEN Biotech. CO., Ltd. (Nanjing, China). Cytotoxicity was determined after 120 min of SN treatment, and the absorbance at 440 nm (reference wavelength was 650 nm) was measured to determine LDH activity. The cytotoxicity to the Caco-2 cell monolayers was expressed by the ratio of LDH released into the supernatant compared with the control group.

The LDH activity was calculated thus: (U/L) = (OD_u_ − OD_c_)/(OD_s_ − OD_b_) × C_s_ × *N* × 1000, where OD_u_, OD_c_, OD_s_, OD_b_ represent the absorbance of the test tube, control tube, standard tube, and background tube, respectively, C represents the standard concentration (2 mmol/L) and *N* represents the dilution ratio of the sample.

#### 3.3.5. Transepithelial Electrical Resistance Measurement and FD-4 Permeability

The cell monolayer integrity was examined by measuring the TEER and evaluating the cell permeability to FD-4 in Caco-2 cell monolayers. The experiments were performed after Caco-2 cells formed polarized monolayers. Cells were seeded onto polycarbonate 12-well Transwell^®^ filter inserts (Corning Costar Corp., Cambridge, MA, USA; pore size 0.4 μm, growth area 1.12 cm^2^) at a density of 1 × 10^6^ cells/mL, and the cells were grown for 21 days before the TEER study was conducted. After the application of 0.5% SN, TEER was measured at time intervals of 20 min for a period of 120 min. Then, the reversibility of SN on TEER was measured by removing SN from the apical compartment and replacing it with serum-free DMEM for a further 120 min at 20-min intervals. To observe the effect of PKC to SN, cells in another group were treated with 0.5% SN and 10 μM Ro318220 (PKC inhibitor) together for 120 min at 20-min intervals. Control experiments were performed under the same conditions but without SN. All of the experiments were performed in triplicate at 37 °C in an atmosphere of 90% relative humidity and 5% CO_2_. The TEER measurements across Caco-2 cell monolayers were performed using a Millicell ERS instrument (Millipore, Bedford, MA, USA). The TEER values of these cells after treatment were recorded. Resistance due to the cell monolayers was determined in the presence and the absence of SN after subtracting the contribution of the blank filter. The percent change in TEER was calculated as follows [19]:

(1) $$\text{TEER}=({\text{R}}_{1}-{\text{R}}_{0})\times \text{A }(\Omega \cdot {\text{cm}}^{2})$$

where R_1_ and R_0_ represent the TEER readings from the wells with cells and the no-cell background wells, respectively, and A (cm^2^) represents the surface area of the cell monolayer on the insert.

(2) $$\text{TEER}\%={\text{TEER}}_{\text{test}}/{\text{EER}}_{\text{initial}}\times 100$$

The integrity of the cell monolayer TJs was also examined by evaluating the FD-4 permeability in PBS adjusted to pH 7.4. A volume of 0.5 mL of 0.1% FD-4 containing 0.5% SN or 0.5% SN + 10 μM Ro318220 was added to the apical side of Caco-2 cell monolayers. The medium in basolateral side was buffered by the addition of 1.5 mL PBS to each well. Samples of 0.1 mL were withdrawn at time intervals of 30, 60, 90 and 120 min from the basolateral side after the incubation of the solutions on the apical side of cell monolayers. The samples withdrawn were replaced with an equal volume of buffered PBS. Control experiments were performed using a solution of 0.1% FD-4 alone. All of the experiments were performed in triplicate at 37 °C in an atmosphere of 90% relative humidity and 5% CO_2_. Basolateral solutions were then sampled and analyzed.

Quantitative analysis of FD-4: Samples were centrifuged at 10,000 rpm for 10 min, and the supernatant was analyzed using a fluorescence spectrophotometer at an excitation wavelength of 495 nm and an emission wavelength of 520 nm (Hitachi, FP6500, Tokyo, Japan).

Permeation clearance [19]: ΔQ/(Δt × C_0_), where ΔQ/Δt is the permeation flux, *i.e.*, the transport rate (μg/min), and C_0_ is the initial concentration on the donor side (μg/mL). Clearance was obtained per unit surface area and expressed as nL/min/cm_2_.

#### 3.3.6. OCT Transport on the Caco-2 Cell Monolayers

Caco-2 cells were seeded onto polycarbonate 12-well Transwell^®^ filter inserts as described above. The OCT transport experiment was performed in the apical-to-basolateral directions in PBS adjusted to pH 7.4 [7]. A volume of 0.5 mL of 0.5% SN containing 10 μM OCT was added to the apical side of Caco-2 cell monolayers. The medium in the basolateral side was buffered by the addition of 1.5 mL PBS of each well. Samples of 0.1 mL were withdrawn at time intervals of 30, 60, 90 and 120 min from the basolateral side after the incubation of the solutions on the apical side of cell monolayers. The samples withdrawn were replaced with an equal volume of buffered PBS. Control experiments were performed using a solution of OCT alone. All of the experiments were performed in triplicate at 37 °C in an atmosphere of 90% relative humidity and 5% CO_2_. The samples were stored at −20 °C until LC-MS/MS analysis.

The results were corrected for dilution and plotted as the percentage cumulative OCT transported as a function of time. The apparent permeability coefficients (*Papp*) were calculated according to the following equation [33]: *Papp* = ΔQ/(Δt × A × C_0_) (cm/s), where ΔQ/Δt is the amount of OCT transported within a given time period or permeability rate (ng/min), A is the surface area of the cell monolayer on the insert (cm^2^) and C_0_ is the initial drug concentration (ng/mL). Permeation enhancement ratios (R) were calculated from the Papp values using the following equation *R* = *Papp_test_/Papp_control_*.

#### 3.3.7. Real-Time PCR Analysis

Caco-2 cells were grown on 6-well plates for 21 days and were then treated with SN for 2 h. Then, SN was removed by washing the cells with PBS twice. The cells were grown with new culture medium for another 2 h. To observe the effect of PKC on SN, cells in another group were treated with 0.5% SN and 10 μM Ro318220 together for 2 h. Control experiments were performed under the same conditions but without SN. The gene level expressions induced by SN treatment and after its removal were investigated. Total RNA was extracted following a standard guanidinium phenol-chloroform extraction protocol [34]. The quantity of mRNA was determined by measuring the optical density at 260 nm (A260 nm = 1 for 40 μg/mL RNA), and the purity of mRNA was assessed by determining the ratio of the optical density obtained at 260 and 280 nm (pure RNA: A260 nm/A280 nm = 2.0) using a UV-1206 spectrophotometer (Shimadzu, Tokyo, Japan). The mRNA was then reverse-transcribed into cDNA, according to PrimeScript^®^ RT Master Mix Perfect Real Time purchased from Takara Bio Inc. (Dalian, China). Quantitative real-time PCR was performed using the Applied Biosystems 7500 faster Real-Time PCR System with the SYBR^®^ Premix Ex Taq™ (Tli RNaseH Plus) Master Mix purchased from Takara Bio Inc. (Dalian, China) in triplicate for each sample and each gene. The primers used were as follows (5′-3′): claudin-1 forward, CATGAAGTGCATGAGGTGCTTAGAA; claudin-1 reverse, TGGCCACTAATGTCGCCAGA; β-actin forward, GGAGATTACTGCCCTGGCTCCTA; β-actin reverse, GACTCATCGTACTCCTGCTTGCTG. PCR conditions used were: denaturation at 95 °C for 30 s, followed by 40 cycles of denaturation at 95 °C for 5 s and 30 s at 60 °C for annealing and 30 s at 72 °C for elongation. The results were expressed as the folds of the CT value for the target mRNA to that of the β-actin mRNA.

#### 3.3.8. Western Blotting

Caco-2 cells were grown on 6-well plates and were treated with SN as described above. After treatment, cytosolic, membrane and total protein were extracted following the manufacturer’s instructions of the test kit from Nanjing KeyGEN Biotech. CO., LTD (China). The protocols for western blot analyses have been described previously [35]. The protein concentration was calculated using the BCA Protein Assay Kit from Nanjing KeyGEN Biotech. CO., LTD (China). Equal amounts of protein samples (30 μg protein) were separated onto SDS-polyacrylamide gels (PKC and β-actin were 12% gels, claudin-1 was 15% gels), and the separated proteins were transferred to a PVDF membrane. After blocking in 5% skim milk in Tris-buffered saline containing 0.05% Tween-20 (TTBS), the PVDF membrane was incubated overnight in blocking buffer with diluted primary antibodies: anti-claudin-1 (Abcam, Cambridge, MA, USA, 1:1000 dilution), anti-PKC-α (Beyotime Institute of Biotechnology, China, 1:500 dilution), and anti-β-actin (Zhongshan Goldenbridge Biological Technology, Beijing, China, 1:500 dilution), at 4 °C. Subsequently, the PVDF membrane was washed three times using TTBS, followed by exposure to the secondary antibody: Peroxidase-Conjugated Affinipure Goat Anti-Rabbit and anti-mouse IgG (Biosynthesis Biotechnology, Beijing, China, 1:2000 dilution). The product bands were photographed, and the density of each product band was quantified. The intensity of each signal was corrected using the values obtained from the immunodetection of β-actin, and the relative protein intensity was expressed as folds of the content in the normal group.

#### 3.3.9. Immunofluorescent Staining

Caco-2 cells were seeded onto 6-well plates, grown to confluence and treated with SN as described above. Immunofluorescent staining studies were performed as standard protocols. The treated cells were washed three times with pre-warmed PBS and were then fixed in 4% paraformaldehyde for 10 min at 20 °C. Non-specific binding was blocked in 5% normal goat serum in PBS for 60 min at 37 °C. Subsequently, cells were incubated overnight in primary anti-claudin-1(Abcam, Cambridge, MA, USA) at a concentration of 1:500 at 4°C. On the next day, this procedure was followed by incubation with Alexa-Fluor^®^488-conjugated Affinipure Goat Anti-Rabbit IgG secondary antibody (Zhongshan Goldenbridge Biological Technology, Beijing, China) at 1:400 for 60 min at 37 °C. Cells were viewed using a Leica TCS NT spectral confocal imaging system coupled to a Leica DM IRBE inverted microscope (Leica, Solms, Germany). The lamp intensity and exposure length were kept constant during the image capture to provide accurate information regarding the intensity of staining. The images shown are representative of three independent experiments.

#### 3.3.10. Statistical Analysis

Groups were compared using one-way analysis (ANOVA) of variance with Dunnett’s multiple comparison tests. The two groups were analyzed by using unpaired, two-tailed T-tests. Statistical tests were performed using SPSS software version 17.0 (SPSS Inc., Chicago, IL, USA). The data were represented as the means ± s.d. (*n* ≥ 3), and statistical significance was indicated at a level of *p* < 0.05.

## 4. Conclusions

OCT intestinal absorption is enhanced by SN both *in vivo* and *in vitro*. Transmembrane protein claudin-1 plays an important role in SN-mediated transient and reversible epithelial TJs opening, which is, at least in part, related to SN-mediated PKC activation.

## Figures and Tables

**Table 1:** The pharmacokinetics parameters of OCT in rats. OCT after i.v. administration showed a single peak immediately at 10 min (T_max_) with a C_max_ of 307 ng/mL, oral administration of OCT showed a single peak later at 30 min (T_max_) with a C_max_ of 44.3 ng/mL, oral administration of OCT with SN produced an uptake profile at 30 min (T_max_) with a C_max_ of 255 ng/mL.

Group	T_max_ (min)	C_max_ (ng/mL)	AUC (ng/mL/min)	F%	ER
OCT p.o.	30	44.33 ± 6.66	4,023.33 ± 417.14	1.63 ± 0.31	1
OCT + 0.5% SN p.o.	30	255.00 ± 8.72 *	21,268.33 ± 1,136.48 #	8.67 ± 1.73 $	5.3
OCT i.v.	10	307.00 ± 6.00	25,135.83 ± 4,548.65		

* *p*(C_max_ of OCT + SN group *vs*. OCT group) < 0.05,

# *p*(AUC of OCT + SN group *vs*. OCT group) < 0.05,

$ *p*(F% of OCT + SN group *vs*. OCT group) < 0.05.

**Table 2:** Measured value of transepithelial electrical resistance (TEER) on Caco-2 cell monolayers. Control group means the no-cell blankground wells, test group means Caco-2cells wells. The TEER values of test group cells were 746.11 ± 46.57 Ω·cm^2^, this indicated good monolayers integrity which was subjected to experimentation.

TEER reading	1	2	3	4	5	7	TEER (Ω·cm^2^)
Blankgroup	104	107	101	105	106	102	104.17 ± 2.32
cells	760	710	830	760	772	790	746.11 ± 46.57

**Table 3:** The transport ability of SN on OCT (10 uM, A-B) in Caco-2 cells. Control group means 10 μM OCT alone, test group means 0.5% SN + 10 μM OCT. *Papp_test_* was (5.89 ± 0.038) × 10^−6^ cm/s, *Papp_control_* was (2.67 ± 0.015) × 10^−6^ cm/s, ^*^p(*Papp_test_vs. Papp_control_*) < 0.05, *R* = 2.2.

Group	*Papp* (×10^−6^ cm/s)	*R (Papp_test_/Papp_control_)*
Control (10 μM OCT)	2.67 ± 0.015	1
0.5% SN + 10 μM OCT	5.89 ± 0.038 ^*^	2.2
